# Optic radiations representing different eccentricities age differently

**DOI:** 10.1002/hbm.26267

**Published:** 2023-03-10

**Authors:** John Kruper, Noah C. Benson, Sendy Caffarra, Julia Owen, Yue Wu, Aaron Y. Lee, Cecilia S. Lee, Jason D. Yeatman, Ariel Rokem, UK Biobank Eye and Vision Consortium

**Affiliations:** ^1^ Department of Psychology University of Washington Seattle Washington USA; ^2^ eScience Institute University of Washington Seattle Washington USA; ^3^ Graduate School of Education, Stanford University and Division of Developmental‐Behavioral Pediatrics, Stanford University School of Medicine Stanford University Stanford California USA; ^4^ Department of Biomedical, Metabolic and Neural Sciences University of Modena and Reggio Emilia Modena Italy; ^5^ Department of Ophthalmology University of Washington Seattle Washington USA; ^6^ Roger and Angie Karalis Johnson Retina Center University of Washington Seattle Washington USA

**Keywords:** aging, diffusion MRI, DKI, optic radiation, retinal mapping, tractography, tractometry, U.K. biobank, visual system

## Abstract

The neural pathways that carry information from the foveal, macular, and peripheral visual fields have distinct biological properties. The optic radiations (OR) carry foveal and peripheral information from the thalamus to the primary visual cortex (V1) through adjacent but separate pathways in the white matter. Here, we perform white matter tractometry using pyAFQ on a large sample of diffusion MRI (dMRI) data from subjects with healthy vision in the U.K. Biobank dataset (UKBB; *N* = 5382; age 45–81). We use pyAFQ to characterize white matter tissue properties in parts of the OR that transmit information about the foveal, macular, and peripheral visual fields, and to characterize the changes in these tissue properties with age. We find that (1) independent of age there is higher fractional anisotropy, lower mean diffusivity, and higher mean kurtosis in the foveal and macular OR than in peripheral OR, consistent with denser, more organized nerve fiber populations in foveal/parafoveal pathways, and (2) age is associated with increased diffusivity and decreased anisotropy and kurtosis, consistent with decreased density and tissue organization with aging. However, anisotropy in foveal OR decreases faster with age than in peripheral OR, while diffusivity increases faster in peripheral OR, suggesting foveal/peri‐foveal OR and peripheral OR differ in how they age.

## Graphical abstract

We performed white matter tractography of the optic radiations (OR) in a large sample of diffusion MRI (dMRI) data from the U.K. Biobank dataset (UKBB; *N* = 6021; age 45–81), dividing each subject's OR into sub‐bundles that represent different parts of the visual field, based on termination sites of different portions of the OR near the visual cortex. Using an advanced model of diffusion (diffusional kurtosis imaging; DKI), we found that (1) independent of age there is higher fractional anisotropy, lower mean diffusivity, and higher mean kurtosis in the foveal and macular OR than in peripheral OR, consistent with denser, more organized nerve fiber populations in foveal/parafoveal pathways, (2) aging is associated with increased diffusivity and decreased anisotropy and kurtosis, consistent with decreased density and tissue organization with aging, and (3) anisotropy in foveal OR decreases faster with age than in peripheral OR, while diffusivity increases faster in peripheral OR, suggesting foveal/peri‐foveal OR and peripheral OR differ in how they age.

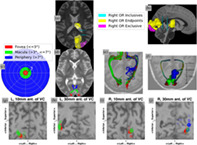

## 1 INTRODUCTION

Visual perception of peripheral and foveal eccentricities differs substantially, suggesting qualitatively different computational mechanisms governing each of these parts of the visual field. These differences in perception and computation stem from structural and functional differences between foveal and peripheral representations at every stage of the visual system. In the retina, the fovea occupies a privileged position with no occlusion by blood vessels or incident axons, and a one‐to‐one ratio of receptors to ganglion cells (Sjöstrand et al., 1999). The magnification of the fovea in the retina is further enhanced as information travels to the cortex, with approximately 50% of the primary visual cortex (V1) devoted to the central 12° of vision (Horton & Hoyt, 1991). Nearby points in the visual field are represented by neighboring points on the surface of the visual cortex (Wandell & Winawer, 2011). This mapping is a consequence of the ordered set of projections from the retina to the lateral geniculate nucleus (LGN) and from the LGN to the V1. The projections from LGN to V1 through the optic radiations (OR), in particular, are known to contain a retinotopic organization, with nerve fibers transmitting information about neighboring parts of the visual field traveling close to each other. Information about nearby points in the visual field travel through adjacent bundles of axons in the white matter, as reflected in the fact that damage to the OR results in scotomas in predictable parts of the visual field (Ebeling & Reulen, 1988). Here, we asked whether there are systematic differences between the white matter projections that transmit information about the central visual field and more peripheral eccentricities.

New large and openly available datasets like the U.K. Biobank (Sudlow et al., 2015) (UKBB) provide an opportunity to study within and cross‐individual differences in the physical properties of the OR at unprecedented scale. Here, we used a large sample (*n* > 5000) from the UKBB dataset to ask whether there are differences in the tissue properties of parts of the OR that contain the axons that transmit information about different parts of the visual field. Furthermore, aging is reflected in measurable changes in the physical properties of the tissue (Cox et al., 2016; Yeatman et al., 2014). In the retina, the fovea has a different trajectory of aging than the periphery (Haas et al., 1986; Heijl et al., 1987). The large range of ages represented in the UKBB sample also allows us to ask whether distinct effects of aging can be measured in different parts of the OR.

To assess white matter tissue properties, we analyzed UKBB diffusion‐weighted MRI (dMRI), which measures the random motion of water within brain tissue (Wandell, 2016). Computational tractography uses directional diffusion information to generate estimates of the trajectories of white matter pathways between different parts of the brain. In the white matter between the LGN and V1, dMRI can be used to accurately delineate the trajectory of the optic radiations (OR) (Caffarra et al., 2021; Kammen et al., 2016; Sherbondy et al., 2008). Furthermore, owing to the systematic mapping of the visual field in the visual cortex, parts of the OR that transmit information about different parts of the visual field can be systematically parsed based on their endpoints close to the visual cortex (Sherbondy et al., 2008; Yoshimine et al., 2018).

In addition to estimating macrostructural white matter tracts, diffusion MRI data can also be used to assess the microstructural properties of the white matter tissue. Here, we used the diffusional kurtosis model (DKI) (Henriques et al., 2021; Jensen et al., 2005). DKI extends the classical diffusion tensor model (DTI (Basser et al., 1994)) by quantifying how much the diffusion deviates from a single Gaussian component. More specifically, low kurtosis indicates a distribution with thinner tails than a Gaussian distribution, and high kurtosis indicates thicker tails. Higher kurtosis indicates heterogeneity in white matter tissue, consistent with more densely packed axons and other cellular structures. Kurtosis is quantified in different directions and is summarized in the mean kurtosis (MK) metric. In addition, we also used metrics from the DTI model, which is  subsumed in the DKI model: the average diffusion across directions (mean diffusivity or MD), and the fractional anisotropy (FA). FA is a normalized variance of the diffusion across directions, which is bounded between 0 (isotropic diffusion) and 1 (anisotropic diffusion). DTI‐derived parameters, such as FA and MD are highly sensitive to biological change and to differences between individuals, but unfortunately, they are also non‐specific. For example, FA tends to decrease with demyelination (Beaulieu et al., 1996), leading some to interpret this parameter as indicative of “white matter integrity”. However, it also decreases in voxels in which more than one major fiber population is present, suggesting that caution should be taken in this interpretation (Jones et al., 2013). In some cases, MK reduces the ambiguity and further constrains the interpretation of FA and MD. For example, both damage to a population of fibers and the addition of crossing fibers reduce FA, but the former causes a decrease in MK, while the latter would cause an increase in MK (Henriques et al., 2021). Tissue properties calculated with DKI also relate to microstructural changes present in brain diseases (Hui et al., 2012; Struyfs et al., 2015).

To assess the tissue properties in different sub‐bundles of the OR, corresponding to different parts of the visual field, and their change with age, we used pyAFQ (https://yeatmanlab.github.io/pyAFQ (Kruper et al., 2021)), an open‐source software pipeline that implements tractometry based on the Automated Fiber Quantification approach (Yeatman et al., 2012). In this approach, white matter pathways, such as the OR, are automatically identified based on anatomical landmarks, and diffusion properties are quantified along the trajectory of the bundle. Tractometry is used to characterize the physical properties of the major white matter pathways along their length, taking into account systematic variability that exists in these properties throughout the length of the major white matter pathways. We recently demonstrated that this process is both reliable in test–retest data, as well as robust to variations in computational methodology (Kruper et al., 2021), despite variability in the results of tractography (Maier‐Hein et al., 2017). Using this approach, we demonstrate a differentiation between white matter properties of different sub‐bundles of the OR, and show that age‐related changes in tissue properties systematically differ between the sub‐bundles. These findings suggest different aging trajectories for different populations of nerve fibers even within the same anatomical pathway.

## 2 METHODS

### 2.1 Data

### 2.2 Subjects

**FIGURE 1:**
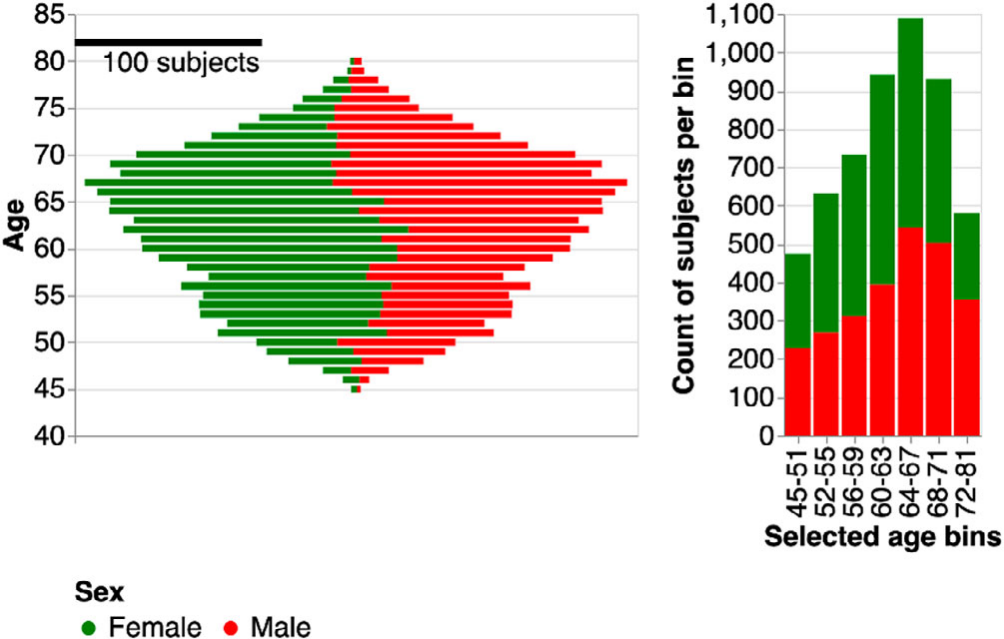
Population characteristics of UKBB subjects. In each panel, gender is denoted by color (green for female, red for male). In the left panel, we plot the age distribution. Note that younger subjects are majority female, while older subjects are majority male. In the right panel, we break down the subjects into age bins. The middle age bins all span 4 years. The first and last age bins were selected to have similar numbers of subjects in each bin to the middle age bins. These age bins are used to group subjects and to visualize changes in tract profiles with age.

We used dMRI data from 7,438 subjects, that were selected because they had both diffusion MRI data and optical coherence tomography measurements in each eye (Sudlow et al. 2015; Alfaro‐Almagro et al. 2018). However, this article focuses only on the dMRI measurements. Of these, we selected 5,382 subjects that are classified as having no eye problems/disorders based on an ACE touchscreen question: “Has a doctor told you that you have any of the following problems with your eyes? (You can select more than one answer)” (see UKBB data field 6148), and who have high visual acuity (logMAR of 0.3 or less). Population characteristics for this sample are shown in Figure 1.

### 2.3 MRI measurements

We used preprocessed diffusion MRI data that were processed and released by the UK Biobank team. The acquisition protocol has been described elsewhere (Miller et al., 2016), and we provide here only some details. Data were acquired with a spatial resolution of 2 × 2 × 2 mm^3^. TE/TR = 14.92/3600 ms. Five volumes were acquired with no diffusion weighting (*b* = 0), and 50 volumes were acquired for each of two diffusion weightings: *b* = 1000 s/mm^2^ and *b* = 2000 s/mm^2^. In addition to these 105 volumes acquired with an anterior‐to‐posterior phase encoding direction, and additional 6 *b* = 0 volumes were acquired with posterior‐to‐anterior phase encoding direction and subsequently used for EPI distortion correction. Preprocessing was also described elsewhere (Alfaro‐Almagro et al., 2018), and we provide only some details. Briefly, head motion and eddy currents were corrected using the FSL “eddy” software, including correction of outlier slices. Subsequently gradient distortion correction was performed. Non‐linear registration using FNIRT was used to map the individual‐level data to the MNI template.

## 3 ANALYSIS

### 3.1 Tractography and registration

**FIGURE 2:**
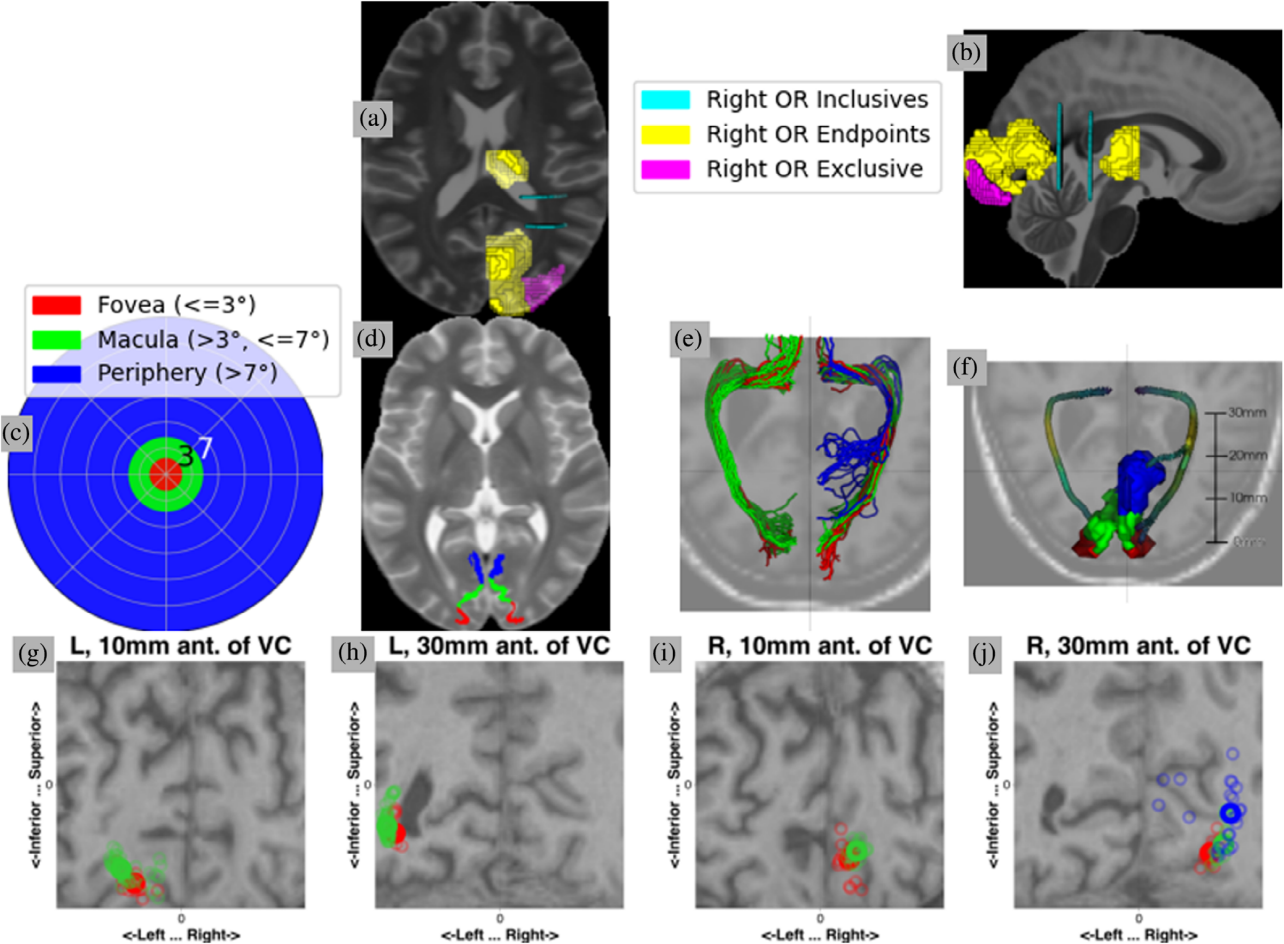
(a,b) Regions of interest (ROIs) for the initial right OR bundle recognition shown over the Montreal Neurological Institute (MNI) T2 template (Fonov et al., 2009). (c) Visual field colored by sub‐bundle divisions. (d) Sub‐bundle divisions shown in V1 over the MNI T2 (Fonov et al., 2009). (e) The streamlines identified in an example subject, colored by sub‐bundle, with T1‐weighted image as background. Note the left peripheral sub‐bundle is not found in this subject. (f) The core bundles used to extract tract profiles in the example subject, with T1 as the background. (g–j) Coronal slices of the T1 along the OR in the example subject. Smaller circles indicate where streamlines pass through the slice, while larger bolded circles indicate where the core bundle passes through the slice. Note that, although the distance between the core bundles may become small (particularly between the macular and foveal sub‐bundles), separation between the sub‐bundles is maintained along the bundle.

Residual bootstrap tractography (Rokem et al., 2021; Berman et al., 2008) was used to delineate the trajectory of optic radiations in each subject's individual data. We used a GPU‐accelerated implementation of this method (Rokem et al., 2021), limiting tractography to the posterior half of the brain. Based on the known trajectory of the optic radiations, we defined inclusion, exclusion, and endpoint regions of interest (ROIs) within the core white matter in each hemisphere (Figure 2a,b). These were registered to each subject's anatomy using the FNIRT non‐linear warp and 64 seeds were uniformly distributed in each voxel of each ROI.

### 3.2 Bundle recognition

Bundle recognition used the pyAFQ software (https://github.com/yeatmanlab/pyAFQ (Kruper et al., 2021)), which implements a procedure very similar to the one described by Yeatman et al. (Yeatman et al., 2012). The software finds streamlines that belong to a white matter pathway by defining waypoint ROIs within the core of the white matter along the trajectory of the pathway. The software allows additional criteria for inclusion or exclusion of tractography streamlines; we used the following criteria to recognize the OR: streamlines (1) do not pass through the sagittal midline of the brain; (2) have at least one point that is within 3 mm of both of the inclusion ROIs; (3) do not have any point that is within 3 mm from the exclusion ROI; (4) terminate within 3 mm of the two endpoint ROIs (one in the thalamus and the other in V1) (Caffarra et al., 2021). After defining this group of streamlines an additional cleaning procedure was applied to remove streamlines that were outliers in terms of their length and trajectory. Subsequently, streamlines were divided into foveal OR (fOR), macular OR (mOR), or peripheral OR (pOR) based on the anatomical position of their termination in V1, using eccentricity from the retinotopic prior of Benson and Winawer (Benson & Winawer, 2018) and masked with the V1 location in the AICHA atlas (Joliot et al., 2015) (Figure 2c–j). The eccentricity ranges were: fOR, ≤3°; mOR, >3°, ≤7°; pOR, >7°.

### 3.3 Tract profiles

The dMRI signal in each voxel was modeled using the diffusional kurtosis model, implemented in DIPY (Garyfallidis et al., 2014; Henriques et al., 2021). The streamlines in each bundle were resampled to 100 points and tissue properties were referred to points along the length of fOR/pOR by extracting the values from the voxels in which each node of each resampled streamline was positioned. Contributions from each node were inversely weighted by their distance from the *core fiber*, the median of the coordinates in each of the 100 nodes along the length of the bundle. In visualizing the results and in statistical analysis, we excluded 20 nodes from either side of the bundle, where tissue properties reflect partial volume effects with the gray matter. An adjusted contrast index (ACI), interpretable as percent difference, is calculated at each position as 2(*x*_1_ − *x*_2_)/(*x*_1_ + *x*_2_) for tissue properties (FA, MD, or MK) *x*_1_ and *x*_2_ from two bundles. The ACI is used to assess differences along a profile. ACIs can be calculated between sub‐bundles or across hemispheres. After calculating ACI and tract profiles for each subject, we display mean profiles/ACI with 95% confidence intervals, calculated using bootstrapping across subjects with 10,000 resamples.

### 3.4 Modeling aging

We modeled aging of tissue properties averaged over all nodes in each bundle. In each bundle, the change of each tissue property over time was modeled as a linear (*a* + *b* × age) and, following Cox et al. (2016), as a quadratic (*a* + *b* × age + *c* × age^2^). Both models were fit using ordinary least squares, implemented in the Statsmodels Python package (Seabold & Perktold, 2010). The two models were compared using Akaike's Information Criterion (AIC). Confidence intervals on model parameters (e.g., *a*, *b*) were estimated using the profile likelihood confidence intervals method (Royston, 2007), as implemented in Statsmodels.

For each tissue property, we also used analysis of variance (ANOVA) to quantify the effects of various predictors on variation in the mean tissue property. We quantify the effects of various predictors on whether a bundle is missing using the same ANOVA setup. The predictors are: age, subbundle, hemisphere, and the interaction between age and subbundle. Because age is continuous, we used an analysis of covariance for that factor. For the sub‐bundle factor and all of its interactions, we used Mauchly's test for sphericity (Mauchly, 1940). When a null hypothesis of sphericity was rejected, the Greenhouse Geisser correction was applied (Greenhouse & Geisser, 1959).

### 3.5 Control bundles

We performed the same analysis on two control bundles, the corticospinal tract (CST) and uncinate (UNC). These bundles were not further divided into subbundles. We used the default ROIs for these bundles as provided in pyAFQ (Kruper et al., 2021).

### 3.6 Software

All code to reproduce the analysis and the figures is available at https://github.com/36000/OR_aging_ukbb.

## 4 RESULTS

**FIGURE 3:**
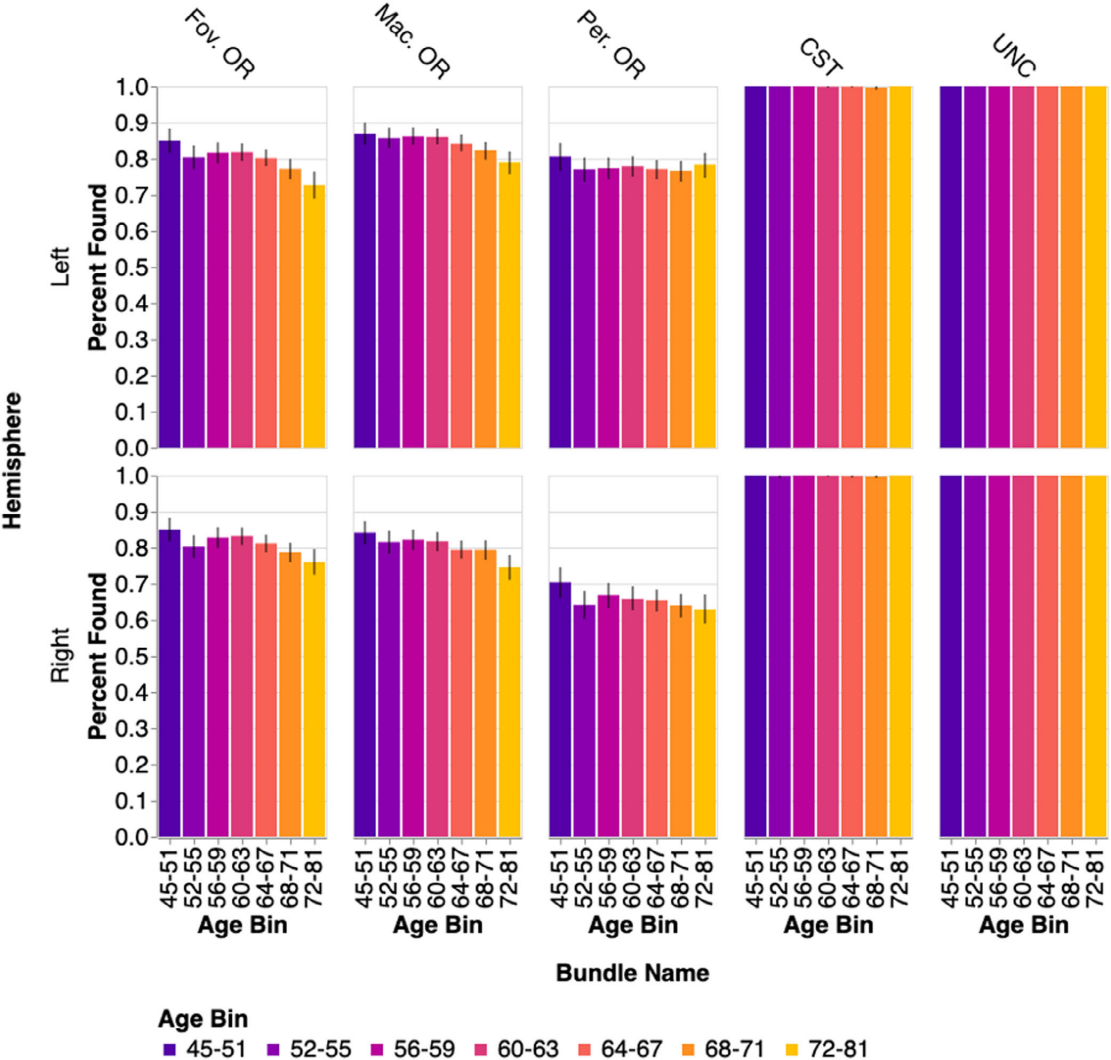
Percentage of successfully delineated bundles by age bin. The control bundles are found in almost all subjects, unlike the OR sub‐bundles, which are harder to track. Note that in some cases, less bundles are found at older ages. Uncertainties show the bootstrapped 95% confidence interval.

We delineated the trajectory of the fOR and pOR in a sample of 5382 subjects from the U.K. Biobank dataset between the ages of 45 and 81. In a portion of these individuals, we were not able to delineate some of the sub‐bundles (Figure 3). Using ANOVA, we find significant main effects of age (*F*_1,5380_ = 18.6, *p* = .00002), sub‐bundle (*F*_1.4,7309.5_ = 342.2, *p* < .00001), and hemisphere (*F*_1,5380_ = 232.3, *p* < .00001). We also find significant interactions between age and subbundle (*F*_1.4,7309.5_ = 6.3, *p* = .00601) but not age and hemisphere (*F*_1,5380_ = 0.01, *p* = .91).

To better understand the effects of the missing bundles, We created a dataset using only subjects where all bundles are found. All 10 bundles are found in only 2,704 subjects. We generated all subsequent visualizations for this dataset, and found the same results. We also ran ANOVA on this smaller dataset, and except where otherwise stated, all significant results in the main 5,382 subject dataset are also significant in the smaller 2,704 subject where all bundles are found.

**FIGURE 4:**
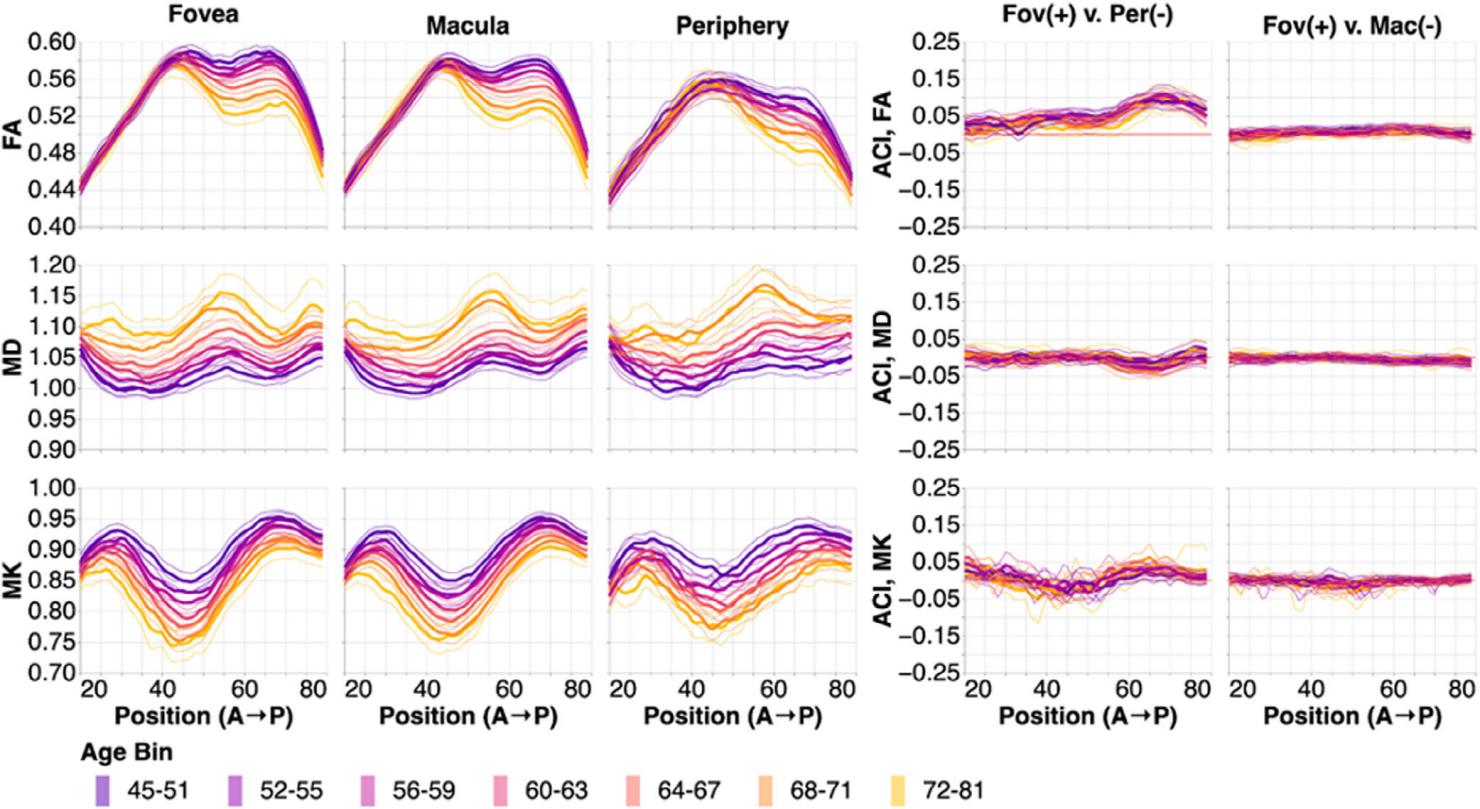
Tissue property profiles along the foveal, macular, and peripheral OR (fOR, mOR, pOR) in the left hemisphere. Positions are from anterior (A) to posterior (P). Subjects are broken down into seven age bins. The first and last age bin have a larger range of ages so that the number of subjects in each age bin are within the same order of magnitude. In the left two columns, tissue properties are plotted by age bins (different line colors: purple is youngest and gold is oldest). Older subjects tend to have lower FA, higher MD, and lower MK. The thin lines show bootstrapped 95% confidence intervals and are also colored according to age bin. In the right column, we show the adjusted contrast index (ACI) between sub‐ORs. Here, higher ACI corresponds to higher values in the tissue properties in the fOR than the pOR or mOR. These differences change only slightly with age, and differences are more pronounced in the posterior section of the OR.

We examine the results in the left hemisphere first (Figure 4). For all tissue properties (Figure 4; rows) and in all sub‐bundles (Figure 4; columns), there are consistent changes in with age across almost the entire profile. FA decreases with age, MD increases with age, and MK decreases with age. We conducted pairwise comparisons between points along each of the bundles using a within‐subject adjusted contrast index, akin to a percent difference. In FA, there are clear differences between fOR and pOR sub‐bundles (the ACI profiles deviate from the red line at 0% difference; Figure 4, under “Fov (+) v. Per (−)”, first row), but it is unclear if there are specific points along the profile where these difference systematically vary with age (different colored lines do not follow a clear gradient). In contrast, there are no points along the profiles, where MK and MD clearly differ between fOR and pOR (the profiles follow the red ACI = 0 line closely; Figure 4, under “Fov (+) v. Per (−),” second and third rows). Similarly, fOR and mOR sub‐bundles do not differ much in any of the tissue properties (the profiles follow the red ACI = 0 line closely; Figure 4, under “Fov (+) v. Mac (−)”).

**FIGURE 5:**
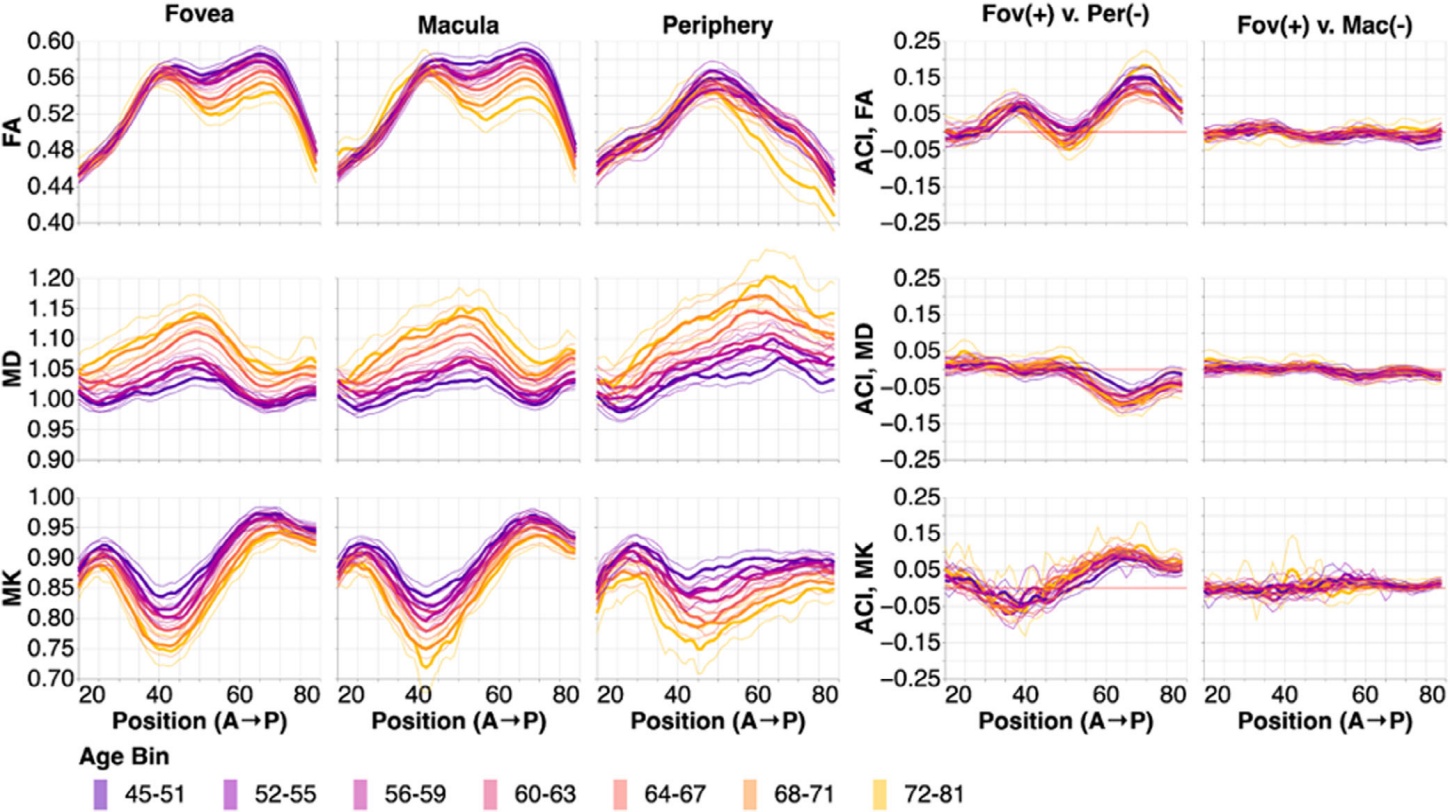
Tissue property profiles along the foveal, macular, and peripheral OR (fOR, mOR, pOR) in the right hemisphere. Positions are from anterior to posterior. Subjects are broken down into seven age bins. The first and last age bin have a larger range of ages so that the number of subjects in each age bin are within the same order of magnitude (see Figure 1). In the left two columns, tissue properties are plotted by age bins (different line colors: purple is youngest and gold is oldest). The thin lines show bootstrapped 95% confidence intervals and are also colored according to age bin. In the right column, we show the adjusted contrast index (ACI) between sub‐ORs. Here, higher ACI corresponds to higher values in the tissue properties in the fOR than the pOR or mOR. These differences change only slightly with age, and differences are more pronounced in the posterior section of the OR.

This pattern of results broadly replicates in the right hemisphere (Figure 5). However, in the right hemisphere, there are distinct differences between fOR and pOR in MK and MD in particular points along their profiles that are not apparent in the left hemisphere (Figure 4).

**FIGURE 6:**
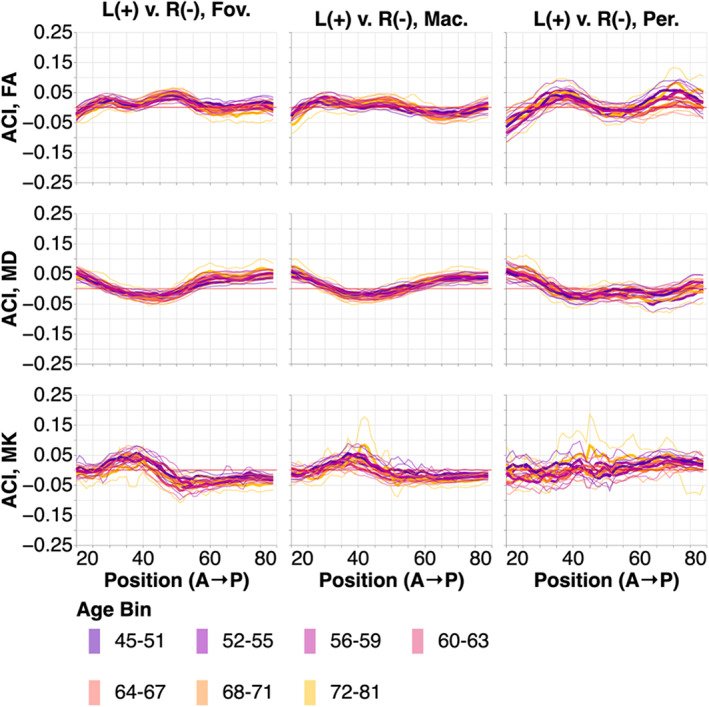
ACI between the left and right ORs. Positions are from anterior to posterior. Subjects are broken down into seven age bins. The thin lines show bootstrapped 95% confidence intervals and are also colored according to age bin. Here, higher ACI corresponds to higher values in the tissue properties in the left OR than the right OR.

In addition to these differences in the patterns of results between the hemispheres, we also find overall differences between the hemispheres, apparent in point‐by‐point comparisons of the right and left hemisphere instance of each sub‐bundle (Figure 6). Even the consistent point‐by‐point differences are generally not very large (almost all ACI are smaller than 5%) and they do not clearly change with age.

**FIGURE 7:**
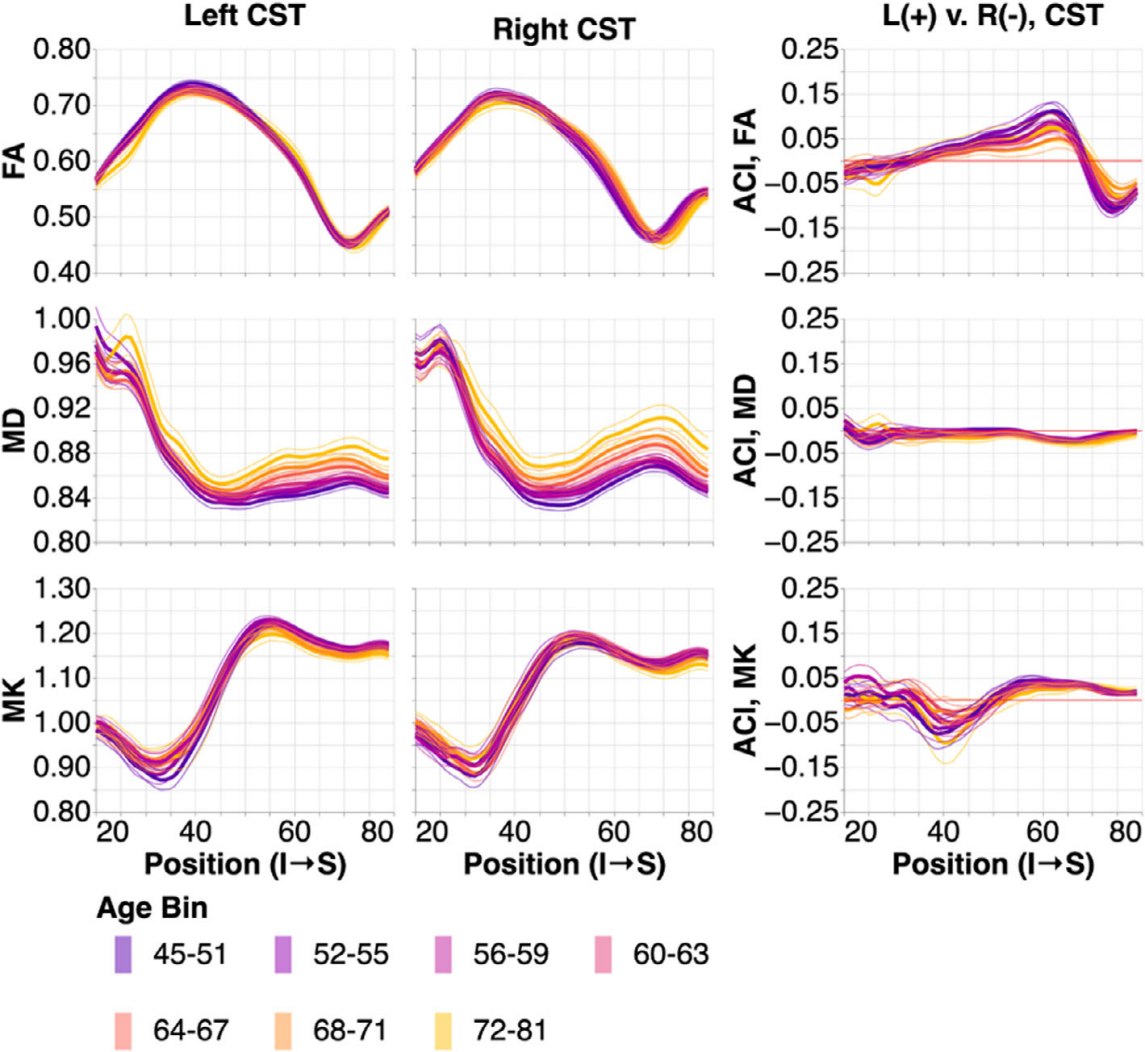
Tissue property profiles along the corticospinal tract (CST). Positions are from inferior to superior. Subjects are broken down into seven age bins. The first and last age bin have a larger range of ages so that the number of subjects in each age bin are within the same order of magnitude. In the left two columns, tissue properties are plotted by age bins (different line colors: purple is youngest and gold is oldest). The thin lines show bootstrapped 95% confidence intervals and are also colored according to age bin. In the right column, we show the adjusted contrast index (ACI) between the left and right CST. Here, higher ACI corresponds to higher values in the tissue properties in the left CST than the right CST.

We compared the tissue properties in the OR to two non‐visual sub‐bundles that we used as a point of comparison in a previous study (Caffarra et al., 2021): the corticospinal tract (CST) and the uncinate fasciculus (UNC). We were not able to delineate left CST in 0.1% of subjects and right CST in 0.1% of subjects. We successfully delineated both UNC in all subjects. Successfully delineated tract profiles and bilateral ACIs are shown in Figures 7 (CST) and 8 (UNC).

In the CST, we see consistent tissue property profile changes with age (Figure 7) that are similar in their directions (FA decrease, MD increase, and MK decrease with age), but smaller in their magnitude than the changes with age in the OR sub‐bundles. CST also has some systematic left–right asymmetries that are particularly large in FA and small in MD. FA profile asymmetries systematically decrease with age.

**FIGURE 8:**
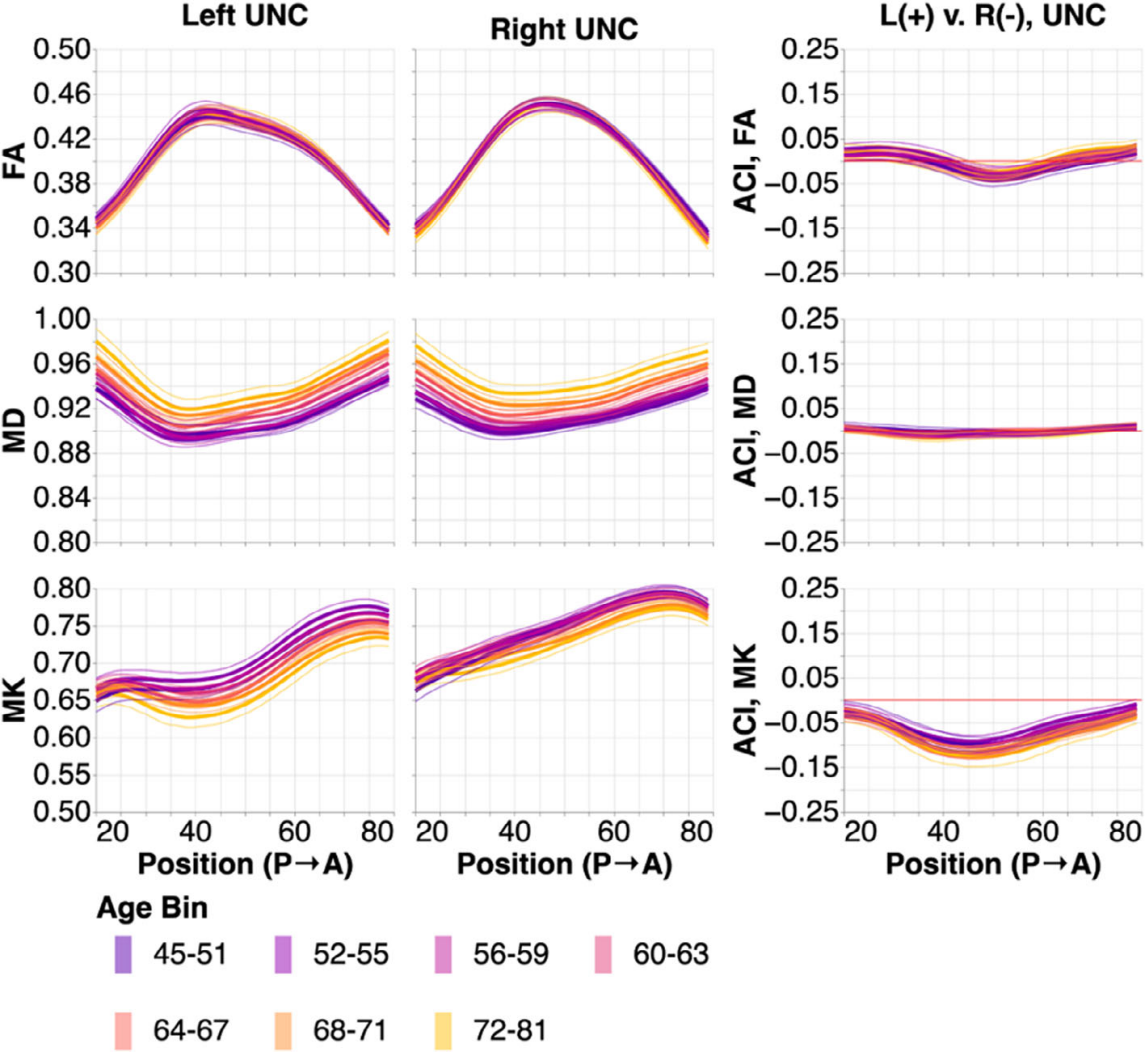
Tissue property profiles along the uncinate (UNC). Positions are from posterior to anterior. Subjects are broken down into seven age bins. The first and last age bin have a larger range of ages so that the number of subjects in each age bin are within the same order of magnitude. In the left two columns, tissue properties are plotted by age bins (different line colors: purple is youngest and gold is oldest). The thin lines show bootstrapped 95% confidence intervals and are also colored according to age bin. In the right column, we show the adjusted contrast index (ACI) between the left and right UNC. Here, higher ACI corresponds to higher values in the tissue properties in the left UNC than the right UNC.

In the UNC (Figure 8), we again observed changes with age that qualitatively resemble the changes we observed in the OR and CST. These changes were smaller than the changes with age in the OR sub‐bundles but larger than the changes observed in CST. Hemispheric asymmetries along the length of the UNC are particularly large in MK and these MK asymmetries increased with age.

To further analyze the tissue properties in the bundles, we averaged each of these quantities along the length of the profiles for every subject and every bundle/sub‐bundle. Analysis of tissue profile means recapitulated some of the results that were observed in the point‐by‐point analysis, and revealed some new observations. For example, in this analysis, we can see that in the CST, mean FA is higher, mean MD is lower, and mean MK is higher than in both the OR sub‐bundles. In UNC, mean FA, mean MD, and mean MK are lower than in the OR sub‐bundles. OR sub‐bundles are similar to each other, but the peripheral OR tends to have lower FA, higher MD, and lower MK.

**FIGURE 9:**
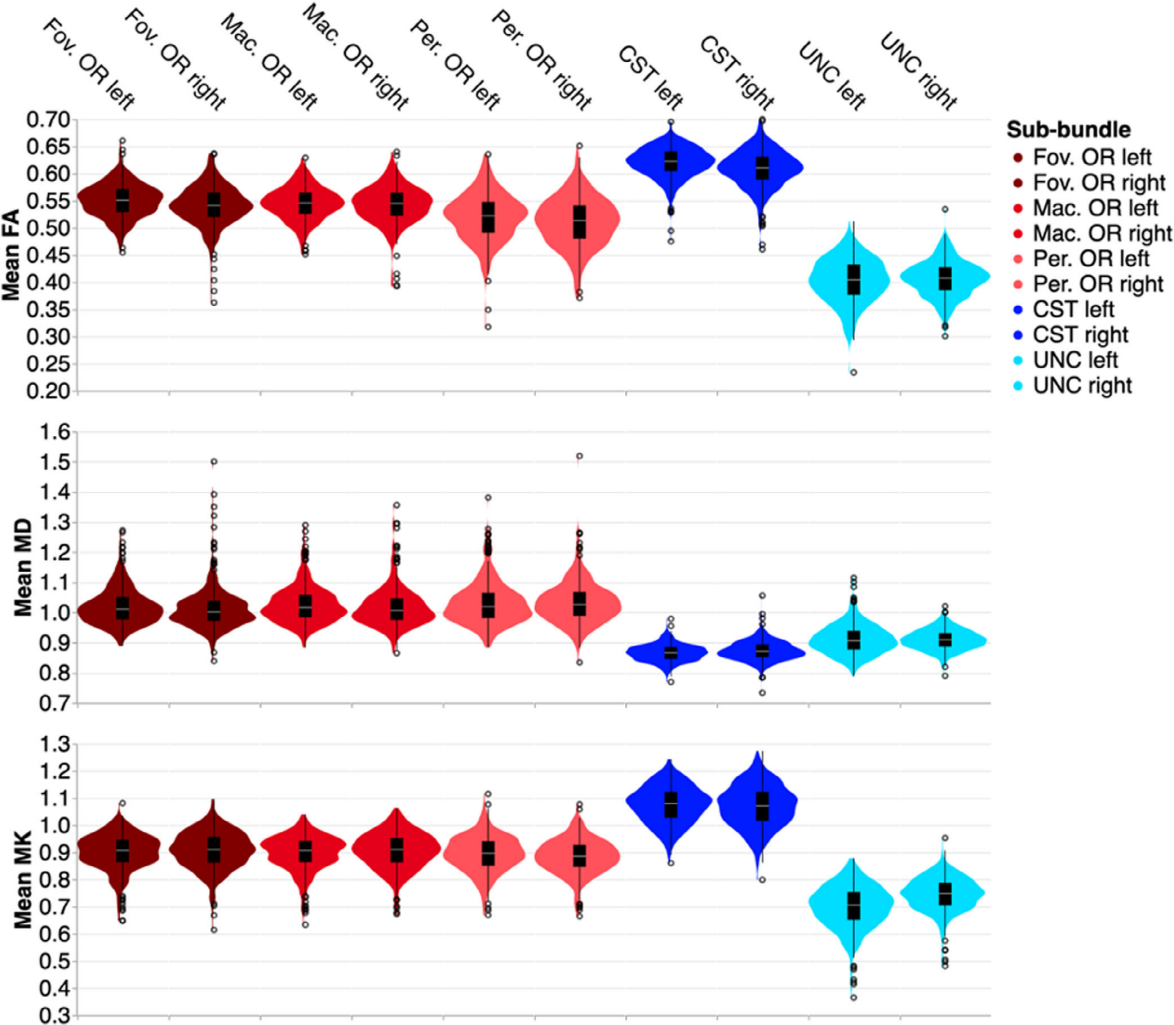
Distribution of mean microstructural tissue properties in the youngest age group (45–51). The wide distributions demonstrate the individual variability in this age group. Note that pOR subbundles have slightly lower mean FA, higher mean MD, and lower mean MK.

Using an ANOVA, we model the averaged tissue properties in the OR sub‐bundles in terms of hemisphere, aging, and sub‐bundle. As expected from the point‐by‐point analysis (Figures 4, 5), we found that mean FA significantly decreases with age (*F*_1,4945_ = 204.5, *p* < .00001). Even while accounting for age, there are also significant differences between the sub‐bundles representing different parts of the visual field (*F*_1.5,7470.8_ = 3678.9, *p* < .00001), presumably because of the lower mean FA in the pOR relative to fOR and mOR (Figure 9). In the aggregate, the small differences seen in the point‐by‐point hemispheric asymmetry analysis do also constitute a significant lateralization effect, with a higher mean FA in the left hemisphere than in the right hemisphere (*F*_1,4945_ = 178.5, *p* < .00001). In addition, the effects of age on FA did not affect all sub‐bundles uniformly, indicated through a significant interaction between age and subbundle (*F*_1.5,7470.8_ = 38.4, *p* < .00001). We explore this effect in more detail below. MD significantly increases with age (*F*_1,4945_ = 622.4, *p* < .00001). We also found a lateralization effect in MD, with higher MD in the left hemisphere than in the right (*F*_1,4945_ = 74.9, *p* < .00001), and a sub‐bundle effect (*F*_1.7,8364.3_ = 512.1, *p* < .00001). Here, interaction between sub‐bundle and age is not significant. MK significantly decreases with age (*F*_1,4945_ = 519.8, *p* < .00001), is significantly higher in the right than in the left hemisphere (*F*_1,4945_ = 272.6, *p* < .00001), and has a sub‐bundle effect (*F*_1.6,7743.9_ = 564.4, *p* < .00001). For MK, an interaction between sub‐bundle and age was found to be marginally significant (*F*_1.6,4269.5_ = 4.7, *p* = .01528) only when considering the subjects for whom all bundles could be delineated.

**FIGURE 10:**
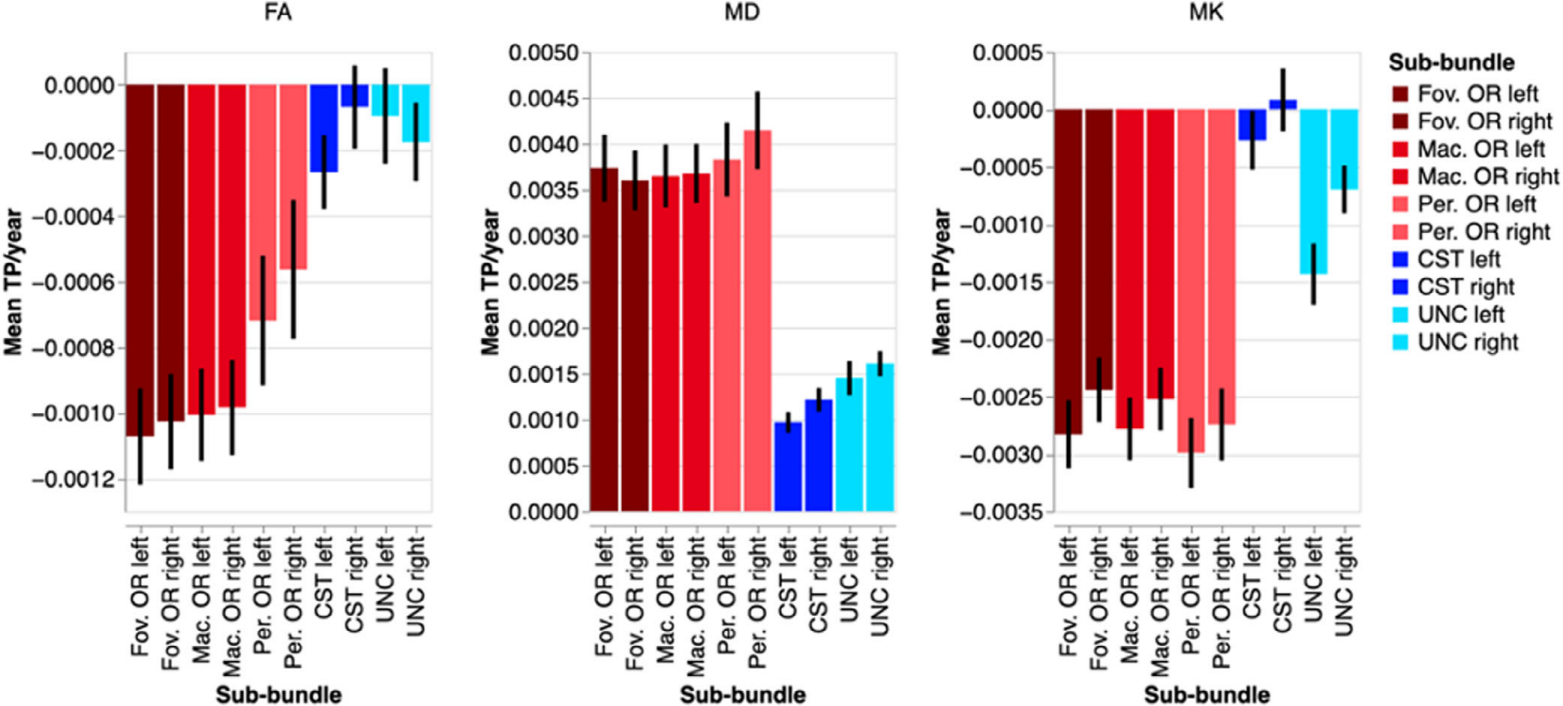
Change in microstructural tissue properties per year according to a linear regression of the mean of each metric. Error bars show the 95% confidence interval. Note that fOR/mOR change more dramatically with age in mean FA than pOR, and that the OR sub‐bundles change more with age than the control bundles.

To further understand the manner in which age affects the averaged tissue properties, we fit separate linear and quadratic regression models to the mean of each tissue property in each bundle and sub‐bundle (Figure 10). Model comparison using AIC found these two models to be closely equivalent in terms of their fit to the data, and we chose to focus on the linear model, because the slope coefficient in this model is more readily interpretable as the average rate of change in a tissue property. OR sub‐bundles all change substantially more rapidly with age than the control bundles, in all three tissue properties, indicated by linear regression slopes of larger magnitudes. The significant age by sub‐bundle ANOVA interaction is explained by the consistent differences between the rate of change in the two central visual field OR sub‐bundles and pOR: FA decreases more rapidly in fOR and mOR than in pOR. Much smaller differences in rate of change are observed in MK and MD. However, MD in the pOR increases at a faster rate than in the fOR and mOR.

## 5 DISCUSSION

Many aspects of brain physiology and structure change with aging. However, aging is not uniform across different parts of the brain, with some regions of the brain more susceptible to aging than others. For example, it is known that different white matter pathways age at different rates (Cox et al., 2016). The goal of the present study was to characterize the aging of the optic radiations, with a particular focus on sub‐bundles of the OR that represent different parts of the visual field. Consistent with results from post‐mortem dissections (Peltier et al., 2006), we found OR sub‐bundles that follow different anatomical trajectories to different parts of the visual cortex. In addition, we found that the microstructural tissue properties in the pOR differ from those measured in the fOR/mOR. We found higher FA, lower MD and higher MK in fOR/mOR relative to pOR. These results are consistent with more densely packed and coherently oriented white matter in the foveal/macular OR relative to the peripheral OR. In this case, the diffusional kurtosis model (DKI) provides additional support to the DTI‐based interpretation (Henriques et al., 2021; Jensen et al., 2005). Importantly, in previous work we have demonstrated that even DTI‐based metrics such as FA and MD are more accurately and reliably estimated using the DKI model, as done here. The differences between the subbundles may relate to information processing differences between more central and more peripheral parts of the visual field. For example, visual processing has much higher acuity in fovea than in periphery (Cowey & Rolls, 1974) and contains more information about color (Hansen et al., 2009), possibly requiring higher information transmission.

Tissue properties of all OR bundles change substantially with age. We modeled age‐dependence of tissue properties using both a quadratic model that has been previously applied to white matter in the UK biobank (Cox et al., 2016), as well as a linear regression model. In our interpretation of these models, we chose to focus on the linear model, because it achieved identical goodness‐of‐fit to the quadratic regression, but its coefficients are easier to interpret as a rate of change in the tissue properties with age. We found that the mean tissue properties in the OR overall age more rapidly than the tissue properties in two control bundles that we analyzed: the corticospinal tract (CST) and the uncinate fasciculus (UNC). The relatively faster aging in OR is consistent with previous results with a smaller sample of UKBB participants (Cox et al., 2016). Nevertheless, it is remarkable that CST tissue properties, and specifically FA, change very little, particularly in an aging population where overall motor abilities also change over time. Importantly, results from tractometry of CST, particularly in the superior part of this pathway, tend to bias towards the representations of the lower body, because of the difficulties to track through the centrum semiovale to the upper body representations that lie more lateral and inferior on the banks of the central sulcus. This is somewhat improved using the probabilistic tractography methods used here, as validated in a patient study that used the same tractography algorithm paired with intraoperative electrical stimulation in brain tumor patients (Mandelli et al., 2014), but is not entirely resolved even with these methods. Regardless, the CST was not the focus of the present study and this result would need further study in populations with different age‐related mobility changes.

Within the OR, we found that all sub‐bundles age in a manner that is consistent with age‐related declines in density and tissue organization (decreased FA, increased MD and decreased MK). However, concomitant faster declines of FA in fOR/mOR and faster increase in MD in the pOR suggest distinct aging processes happening in parallel. Overall, parts of the visual field with higher‐resolution vision (fovea/macula) are associated with white matter bundles that have higher FA, lower MD, and higher MK. Taken together, these two sets of findings are consistent with the high degree of information transmission that needs to be handled by the optic radiations, and particularly within the foveal and macular portions. Replicating previous results (Dayan et al., 2015; Levin et al., 2010; Sherbondy et al., 2008), we also found consistent lateralization effects, with higher FA, higher MD and lower MK in the left than in the right hemisphere.

The study and our conclusions are still subject to several limitations. First, automatically detecting the OR within every individual is a challenging computational task, particularly across a large and diverse sample (e.g., in terms of their ages). This is because of the high curvature of the tract, its narrow path leading into the occipital pole and its intersection with multiple other pathways, challenges which could be compounded by the expansion of the lateral ventricles with age. Moreover, defining the sub‐bundles of the OR is also challenging and we were not able to define the OR bundles or sub‐bundles in many of the subjects (Figure 3). In addition to the large variance in subject ages, this could reflect variable data quality among different individuals in the sample, and reflects the difficulties of consistent tractography to cortical (i.e., V1) and small subcortical (i.e., LGN) targets. To complicate matters, missingness is not randomly distributed with respect to the bundles and the age bins of interest. However, the main conclusions described above hold if we only consider subjects for whom all the bundles could be delineated. Thus, we conclude that the patterns of missingness and bundle‐specific differences in tissue properties are reflecting similar biological processes in terms of the change in the tissue. When FA and MK are lower, and MD is higher, in older subjects (i.e., in the pOR) this bundle is also harder to find because tractography algorithms require directional information to track through these regions of the white matter.

Finally, we rely on participant self‐report, in addition to visual acuity, to exclude participants with eye diseases, and it is possible that some of the results are driven by an increased prevalence of early‐stage undetected eye diseases in older participants (but see also discussion of this point in Mehta et al. (Mehta et al., 2021)). It is possible that this is driving some of the difference between OR and the non‐visual control bundles in this sample. However, even with these challenges, the advantages of the UKBB dataset are clear: it provides a very large sample, providing high confidence in the consistent results that we see here. Finally, tractography cannot differentiate feedforward axons that transmit information from the LGN to cortex from the feedback projections that transmit information in the opposite direction. Though these feedback projections are thought to be abundant (Briggs, 2020), their relative volume fraction within the bundle is not well known. Thus, our conclusions need to be viewed as encompassing the properties of both feedforward and feedback projections within the OR.

To summarize, our findings show that the white matter pathways carrying information from different parts of the visual field have distinct biological properties. The largest differences occur between the pOR and fOR/mOR, which follow different anatomical trajectories. These different sub‐bundles also have different functional properties. The somewhat more rapid decreases in FA in the fOR/mOR may be consistent with the higher degree of information transmission in this part of the visual field. It is also consistent with two related studies that show decrease in surface area representing the central 7° and increased visual population receptive field sizes in this part of V1 and other early visual cortical areas, as measured with functional MRI (Brewer & Barton, 2012; Brewer & Barton, 2014). Hence, the distinct aging of fOR and mPR relative to pOR may also be inherited by other structures further into the visual system. Retinotopic organization of the OR is also apparent in the callosal tracts that connect the visual cortex in both hemispheres (Bock et al., 2013) and a similar analysis could also be applied to the sub‐bundles of the corpus callosum to study this topic further. The methods used here to delineate the different sub‐bundles of the OR could be carried forward into population studies of visual disorders that differentially affect different parts of the visual field, such as age‐related macular degeneration, as has already been done in small samples (Yoshimine et al., 2018). More generally, the findings demonstrate consistent anatomical variation in tissue properties and their aging even within a single white matter pathway.

## CONFLICT OF INTEREST STATEMENT

Dr. A. Lee reports grants from Santen, personal fees from Genentech, personal fees from US FDA, personal fees from Johnson and Johnson, grants from Carl Zeiss Meditec, personal fees from Topcon, personal fees from Gyroscope, non‐financial support from Microsoft, grants from Regeneron, outside the submitted work; This article does not reflect the views of the US FDA.

## Data Availability

This study uses publicly available data from the UK Biobank. More information on the data and access can be found here: https://www.ukbiobank.ac.uk/enable-your-research.
